# Effect of *trans*-Cinnamaldehyde-Loaded ZIF‑8 Nanoparticles against *Escherichia coli* MG1655 Adhered to Stainless-Steel, Polyurethane, and Latex Surfaces

**DOI:** 10.1021/acsomega.5c12189

**Published:** 2026-07-08

**Authors:** Zeynep Sevimli-Yurttas, Rosana G. Moreira, Elena Castell-Perez

**Affiliations:** Department of Biological and Agricultural Engineering, 14736 Texas A&M University, College Station, Texas 77843-2117, United States

## Abstract

This study evaluated the effectiveness of *trans*-cinnamaldehyde-entrapped Zeolitic Imidazole Framework-8 (ZIF-8) (TC@ZIF-8) nanoparticles in removing biofilms of *Escherichia coli* MG1655 adhered to common food-contact surfaces. *trans-*Cinnamaldehyde (TC) release was tested at room temperature using a phosphate-buffered saline (PBS): methanol (5%) mixture and UV–visible spectrophotometry at 295 nm. 1 cm × 1 cm coupons made from stainless steel, polyurethane sheet, and latex were characterized in terms of surface free energy and contact angles. The surface morphology of the different coupons was analyzed using scanning electron microscopy (SEM). Antibacterial activity was assessed by determining the minimum inhibitory concentration (MIC) and the minimum bactericidal concentration (MBC). The ability of the 0.5TC@ZIF-8 nanoparticles to prevent the growth of *E. coli* MG1655 colonies on the coupons and to remove already established *E. coli* MG1655 biofilms was evaluated at different MBC concentrations. A 2×MBC concentration prevented bacterial attachment on stainless steel and latex surfaces. In contrast, a 20×MBC concentration with 24 h contact time was required to remove already established biofilms from the stainless-steel surface. The same treatment yielded a 1.88-log and a 3.38-log reduction on polyurethane and latex surfaces, respectively.

## Graphical abstract

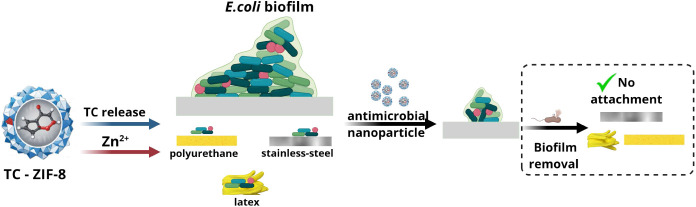

## Introduction

Microbial biofilms arise when microorganisms irreversibly attach to solid interfaces and produce extracellular polymeric substances that enhance persistence and resistance to cleaning and disinfection strategies.^1,2^ In food processing and handling environments, these structured microbial communities frequently develop on surfaces such as pipelines, conveyors, packaging materials, and utensils, where they can act as continuous sources of cross-contamination.^3,4^ Once established, biofilms may negatively affect product shelf life, contribute to foodborne disease outbreaks, and impair equipment performance through corrosion, clogging, and reduced heat transfer efficiency.^2,4^

The initial stages of biofilm development are strongly influenced by the physicochemical characteristics of the contact surface. Parameters such as surface roughness, surface free energy, and wettability govern bacterial adhesion forces and attachment probability.^5,6^ Surfaces exhibiting higher surface free energy or greater wettability have been associated with enhanced microbial retention,^7,8^ while surface topography may either facilitate or hinder attachment, depending on scale and morphology.^5,9^ Consequently, evaluating antimicrobial interventions across materials with distinct surface properties is essential for accurately assessing biofilm control strategies in practical settings.

Conventional sanitation practices in the food industry rely heavily on chemical disinfectants; however, concerns regarding the formation of toxic byproducts, environmental impact, and the emergence of antimicrobial resistance have driven interest in naturally derived alternatives.^10^ Essential oils and their active components have attracted attention due to their broad-spectrum antimicrobial activity.^11^ Among these compounds, *trans*-cinnamaldehyde, a major constituent of Cinnamomum cassia bark oil, has demonstrated effectiveness against a wide range of bacteria, molds, and yeasts.^12–17^ Despite its antimicrobial potency, the application of *trans*-cinnamaldehyde is limited by its volatility, poor aqueous solubility, and reduced stability during prolonged exposure.^18^

Nanostructured delivery systems offer opportunities to overcome these limitations by improving stability and enabling controlled release of bioactive compounds. Zeolitic imidazolate framework-8 (ZIF-8), a metal–organic framework composed of zinc ions and imidazolate linkers, has been explored as a carrier system due to its high surface area, tunable porosity, and compatibility with antimicrobial applications.^19^ Encapsulation of *trans*-cinnamaldehyde within ZIF-8 can reduce volatilization, improve dispersion in aqueous systems, and support sustained antimicrobial activity. In addition, partial degradation of the framework under aqueous conditions may release Zn^2+^ ions, further contributing to antibacterial performance.^19,20^

*Escherichia coli* MG1655, a laboratory strain derived from *E. coli* K-12, was selected as the model microorganism due to its documented ability to form dense and mature biofilms within 24 h.^21–23^ Compared to other *E. coli* strains, MG1655 produces higher biofilm biomass during maturation, making it suitable for evaluating surface-associated antimicrobial interventions.

While the synthesis and physicochemical characterization of *trans*-cinnamaldehyde-loaded ZIF-8 nanoparticles have been previously reported^24^ their performance against biofilms adhered to food-contact materials with differing surface energies and roughness profiles has not been systematically evaluated. The objective of the present study was therefore to assess the effectiveness of TC@ZIF-8 nanoparticles in preventing bacterial attachment and removing established *E. coli* MG1655 biofilms from stainless steel, polyurethane, and latex surfaces.

## Materials and Methods

### Materials

Zinc nitrate hexahydrate (Zn­(NO_3_)­2·6H_2_O), 2-methylimidazole, and *trans*-cinnamaldehyde (TC) were used for nanoparticle synthesis.^22,23^ Culture media, solvents, buffer components, and analytical reagents were obtained from commercial suppliers and used without further purification, as previously described.^23^

### Food-Contact Surface Materials and Coupon Preparation

Food-contact materials included AISI 304 stainless steel, polyurethane sheet material, and disposable natural latex gloves, selected to represent surfaces common in food processing and handling environments.^1,3^ Coupons (1 × 1 cm) were prepared from each material. Stainless steel coupons were washed with laboratory detergent, rinsed, dried at 60 °C, autoclaved, and stored aseptically prior to use.^17^ Polyurethane and latex coupons were washed, disinfected using a chlorine solution, dried overnight in a biosafety cabinet, exposed to UV radiation on both sides, and stored in sterile containers until further analysis.^3,34^

### Synthesis of TC@ZIF-8 Nanoparticles

TC-loaded ZIF-8 nanoparticles were synthesized using a one-pot coordination method adapted from previously reported protocols.^22,23^ Zinc nitrate hexahydrate and 2-methylimidazole were dissolved separately in ethanol, and TC was incorporated into the ligand solution prior to mixing. The solutions were combined under ultrasonic agitation, followed by centrifugation, repeated washing, and vacuum drying to obtain the final nanoparticles.^23^

### Characterization of Contact Surfaces (Coupons)

#### Contact Angle and Surface Free Energy

Static contact angle measurements were used to evaluate surface wettability of the coupon materials.^24,25^ Droplets of test liquids were placed on coupon surfaces and imaged for analysis. Surface free energy was calculated using the Owens–Wendt–Rabel–Kaelble (OWRK) model based on contact angle measurements obtained with polar and nonpolar probe liquids.^26–29^

### Surface Roughness Characterization with Scanning Electron Microscopy

Surface morphology and relative roughness of the coupons were examined using scanning electron microscopy (SEM) with a Tescan Vega SEM (RRID: SCR_022128) operated at 20 kV and a working distance of 9–15 mm. Prior to imaging, coupons were sputter-coated with a thin conductive metal layer. Micrographs were collected at multiple magnifications to qualitatively assess surface topography.

### Characterization of 0.5TC@ZIF-8 Nanoparticles

The structural and physicochemical characterization of ZIF-8 and TC@ZIF-8 nanoparticles, including XRD, FTIR, particle size, morphology, and encapsulation efficiency, was previously reported by ref 23 and is therefore not repeated here. Based on that, the 0.5TC@ZIF-8 nanoparticles were selected for the present study as the most effective combination. Therefore, only the release of TC from the particles and the assessment of antibacterial effectiveness are reported in the present study.

### TC Release

Release behavior of TC from ZIF-8 nanoparticles was evaluated in phosphate-buffered saline containing 5% methanol at 19 and 37 °C under constant agitation.^23,30^

### Antibacterial Activity

Direct comparison with free *trans*-cinnamaldehyde was not the focus of this study, as its rapid volatility and limited aqueous stability restrict its applicability for prolonged surface contact. The objective here was to evaluate the effectiveness of a stabilized delivery system under conditions relevant to surface-based biofilm control.

### Microorganism and Culture Preparation

*Escherichia coli* MG1655 was used as the test organism due to its well-documented biofilm-forming capability.^19–21^ Frozen stock cultures were revived in tryptic soy broth and incubated aerobically at 37 °C. Working cultures were prepared from isolated colonies and maintained at 4 °C for short-term use following biosafety level 2 procedures.^30^

### Determination of Minimum Inhibitory and Bactericidal Concentrations

The minimum inhibitory concentration (MIC) and minimum bactericidal concentration (MBC) of TC@ZIF-8 nanoparticles were determined using a broth microdilution method as previously described.^31–33^ Growth inhibition was monitored spectrophotometrically, and bactericidal activity was confirmed by plate enumeration.^32^ The concentrations that showed 3-log reduction on the plate surfaces following incubation were considered bactericidal. The lowest concentration of nanoparticles demonstrating bactericidal activity across all replicates was considered the minimum bactericidal concentration (MBC).^33^

After incubation, coupons were removed aseptically and gently rinsed three times with sterile distilled water to remove loosely attached and planktonic cells. Excess liquid on the coupon surfaces was carefully removed using sterile absorbent paper prior to further processing.

### Biofilm Formation, Prevention, and Removal Experimental Design

Biofilm experiments were conducted using four distinct treatment schemes: untreated controls, solvent controls, biofilm prevention assays, and biofilm removal assays (Figure 1).

**Figure 1:**
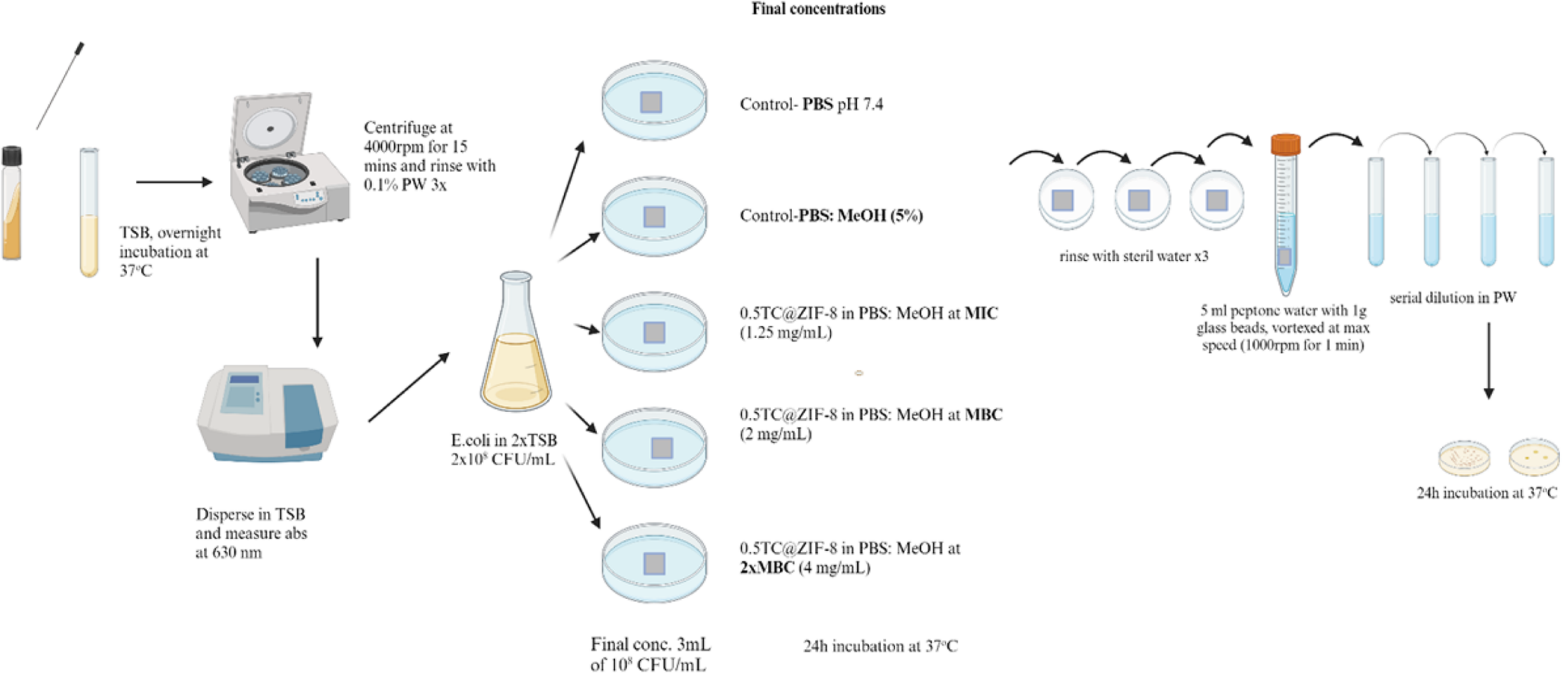
Bacterial attachment and inhibition of growth of *E. coli* MG1655 in the presence of 0.5TC@ZIF-8 nanoparticles (created with BioRender.com).

#### (i) Untreated and Solvent Controls

For control experiments, stainless steel, polyurethane, and latex coupons were incubated with *Escherichia coli* MG1655 suspensions without nanoparticles (untreated control) or in phosphate-buffered saline (PBS) containing 5% methanol (solvent control). These controls were included to account for any effects associated with the carrier solution and were processed identically to treated samples during recovery and enumeration procedures.^34,35^

#### (ii) Biofilm Formation (Baseline Attachment)

Baseline biofilm formation was achieved by submerging prepared coupons in 30 mL of *E. coli* MG1655 suspensions adjusted to approximately 1 × 10^8^ CFU/mL and incubating under static conditions at 37 °C for 24 h to allow bacterial attachment and early biofilm development.^34,35^ Following incubation, coupons were gently rinsed three times with sterile distilled water to remove loosely attached and planktonic cells. Excess surface liquid was removed using sterile absorbent paper prior to further processing.

#### (iii) Biofilm Prevention Assays

To evaluate prevention of biofilm formation, TC@ZIF-8 nanoparticle suspensions were added directly to the bacterial growth medium at the beginning of incubation at concentrations corresponding to the minimum inhibitory concentration (MIC), minimum bactericidal concentration (MBC), and two times the MBC (2 × MBC). Coupons were incubated in these suspensions for 24 h under the same conditions used for baseline biofilm formation. After incubation, coupons were rinsed and processed for bacterial recovery and enumeration using the same protocol applied to control samples.^34,35^

#### (iv) Biofilm Removal Assays

For biofilm removal experiments, 24-h-old preformed biofilms were first established as described above. Coupons were then exposed to TC@ZIF-8 nanoparticle solutions prepared in PBS containing 5% methanol at concentrations up to 20 × MBC. Treatments were applied for contact times of 1 and 24 h at room temperature under static conditions.^34^ To evaluate the effect of mechanical action, selected treatments included vortexing the coupons in the nanoparticle solution in the presence of sterile glass beads (500 μm diameter) to simulate a scrubbing effect commonly applied during sanitation procedures.

### Recovery and Enumeration of Biofilm Cells

The workflow used for biofilm removal, cell recovery, and bacterial enumeration is illustrated schematically in Figure 2. Briefly, following treatment, coupons were aseptically transferred to sterile centrifuge tubes containing 0.1% peptone water and sterile glass beads (500 μm diameter). Biofilm cells were detached from coupon surfaces by vortexing at high speed for a fixed duration to ensure reproducible removal of adhered cells.^34–36^ The resulting suspensions were serially diluted in 0.1% peptone water, and aliquots were spread-plated onto tryptic soy agar plates. Plates were incubated at 37 °C for 24 h prior to colony enumeration. Bacterial populations were calculated and expressed as log_10_ CFU/cm^2^ based on the total exposed surface area of each coupon.^35^

**Figure 2:**
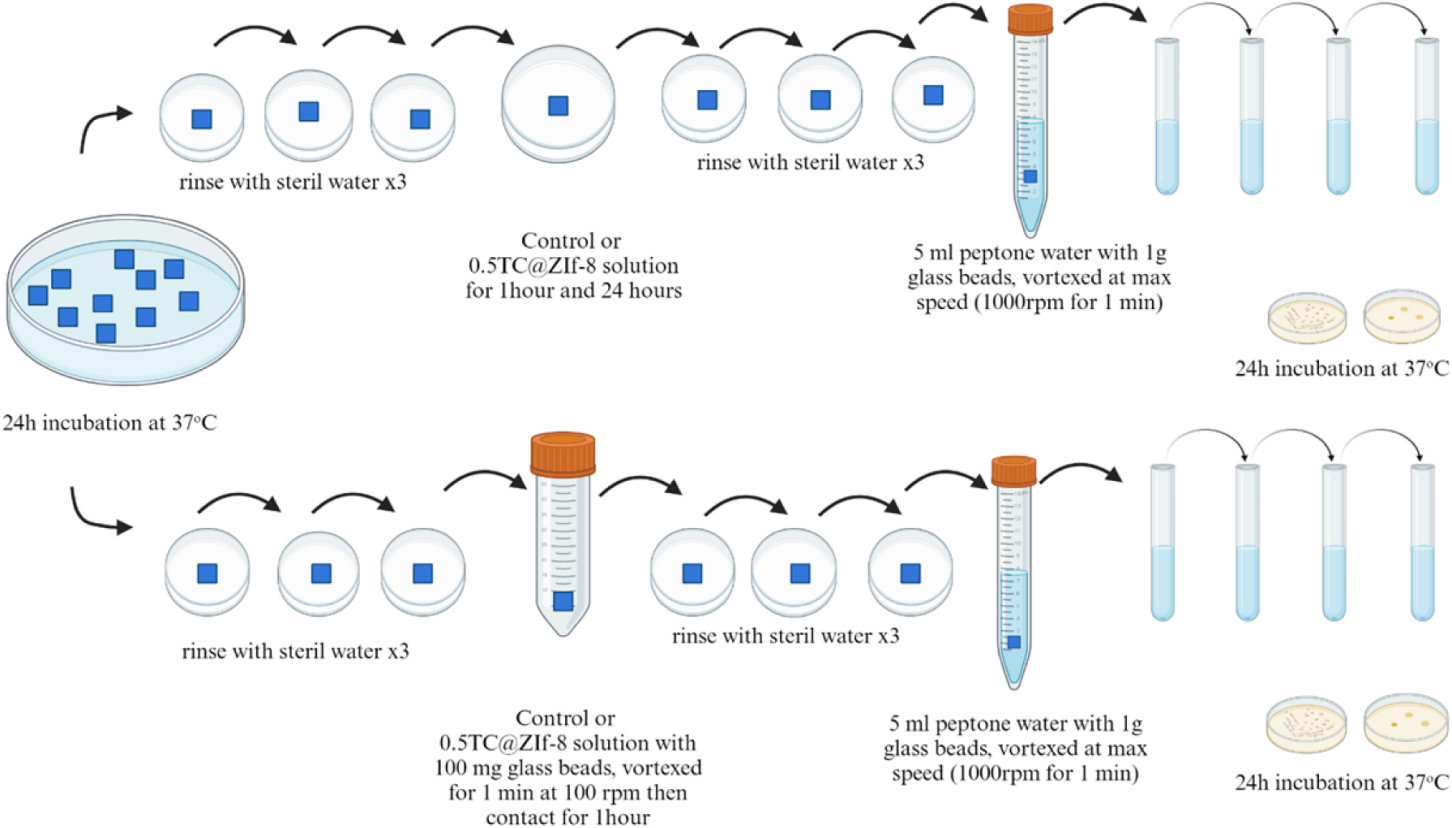
Biofilm removal and bacterial enumeration procedure (created with BioRender.com).

### Statistical Analysis

All experiments were conducted in triplicate, unless stated otherwise. Data were presented as mean values ± standard deviation (SD). Statistical analysis was carried out using SPSS software (version 29.0.0.0.0, IBM SPSS Inc., Armonk, NY, USA). One-way analysis of variance (ANOVA) with Tukey’s multiple range test was used to assess statistical differences, and Student’s *t* test was applied for comparisons between two independent means. Statistical significance was considered at *P* < 0.05.^40^

## Results and Discussion

### Surface Wettability of Food-Contact Materials

The wettability of stainless steel, polyurethane, and latex coupons was evaluated by measuring static water contact angles, with results summarized in Table 1. Distinct differences in wettability were observed among the three materials. Latex exhibited the lowest water contact angle, indicating a highly hydrophilic surface, whereas stainless steel and polyurethane showed significantly higher contact angles and comparatively lower wettability.^37–39,43,44^

**Table 1:** Contact Angle Measurement of Water and 0.5TC@ZIF-8 Nanoparticle Solution on the Three Types of Surfaces^I,II^

Surface type	Water^a^	0.5TC@ZIF-8^a^
Stainless steel	_x_ 75.91 ± 2.98^b^	_x_ 68.23 ± 7.40^a^
Polyurethane	_x_ 76.46 ± 4.72^b^	_x_ 71.46 ± 3.55^a^
Latex	_x_ 61.71 ± 2.75^a^	_y_ 70.09 ± 0.14^a^

a Values given are averages of three replicate samples ± standard deviations.

b Means within a column, which are not followed by a common superscript letter, are significantly different (*p* < 0.05)^.x,y^ Means within a row, which are not followed by a common subscript letter, are significantly different (*p* < 0.05).

Surface wettability is an important parameter governing initial microbial adhesion, as it influences liquid spreading and the extent of surface–cell contact. Hydrophilic surfaces promote greater interaction between the aqueous bacterial suspension and the solid surface, which can enhance bacterial attachment during early colonization stages.^44^ The increased hydrophilicity observed for latex surfaces (Table 1) therefore provides an initial indication of greater susceptibility to bacterial attachment compared to stainless steel and polyurethane surfaces.

In addition to water wettability, the contact angles of the 0.5TC@ZIF-8 nanoparticle suspension on each surface were determined to assess how the antimicrobial formulation interacted with the different materials. These values are also reported in Table 1, and representative droplet images are shown in Figure 3. For stainless steel and polyurethane, the contact angles measured for the 0.5TC@ZIF-8 suspension were slightly lower than those measured for water, although the differences were not statistically significant. This suggests that the presence of the nanoparticle suspension did not substantially alter the inherent wettability of these surfaces.

**Figure 3:**
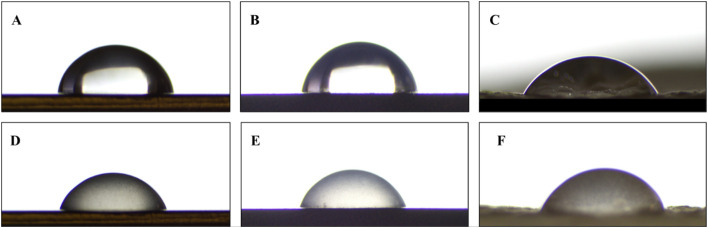
Digital images of water droplets on (A) stainless steel, (B) polyurethane, (C) latex, and 0.5 TC@ZIF-8 solution at MBC concentration on (D) stainless steel, (E) polyurethane, and (F) latex.

In contrast, latex surfaces exhibited a significantly higher contact angle when exposed to the 0.5TC@ZIF-8 suspension compared to water (Table 1 and Figure 3). This increase indicates reduced spreading of the nanoparticle suspension on latex surfaces and suggests altered interactions between the polymeric substrate and components of the suspension. This behavior suggests that interactions between the TC@ZIF-8 formulation and the latex surface differ from those occurring with water alone and may influence antimicrobial contact efficiency during surface treatments.

Despite this reduced wettability, latex surfaces later exhibited high levels of bacterial attachment and biofilm formation, indicating that wettability alone is not sufficient to predict microbial behavior and that additional surface properties must be considered.

### Surface Free Energy of Coupon Materials

To further characterize surface physicochemical properties, total surface free energy values for stainless steel, polyurethane, and latex coupons were calculated using the Owens–Wendt–Rabel–Kaelble (OWRK) approach. The resulting values are presented in Table 2. Among the tested materials, latex exhibited the highest total surface free energy, followed by stainless steel, while polyurethane showed the lowest values. The OWRK model accounts for both polar and dispersive interactions that influence surface affinity and wettability.^26–29,41,42^ These differences indicate substantial variation in surface interaction potential among the tested materials.

**Table 2:** Total Surface Free Energy (mN/m) of coupons

Material	Total surface free energy^a^ [mN/m]
Stainless-steel	36.637 ± 1.245^b^
Polyurethane	30.981 ± 2.583^a^
Latex	45.206 ± 1.490^c^

a Values given are averages of three replicate samples ± standard deviations.

b Means within a column, which are not followed by a common superscript letter, are significantly different (*p* < 0.05).

An inverse relationship between water contact angle (Table 1) and total surface free energy (Table 2) was observed across the three materials, further confirming the complementary nature of wettability and surface energy measurements. Materials exhibiting lower contact angles displayed higher total surface free energy, whereas materials with higher contact angles showed reduced surface energy values. These trends are consistent with the theoretical framework underlying the OWRK model.^26–29^

Surfaces with higher surface free energy have been reported to exhibit stronger interaction forces with bacterial cells, which can enhance microbial adhesion and promote biofilm development.^44^ In the present study, the elevated surface free energy measured for latex coupons is consistent with the higher levels of bacterial attachment observed during biofilm formation experiments. Conversely, polyurethane, which exhibited the lowest surface free energy, showed reduced bacterial attachment under comparable conditions. Stainless steel displayed intermediate behavior, reflecting its moderate surface free energy relative to the polymeric materials.

These physicochemical differences provide essential context for the surface morphology analysis presented in Figure 4 and for interpreting material-dependent biofilm behavior.

**Figure 4:**
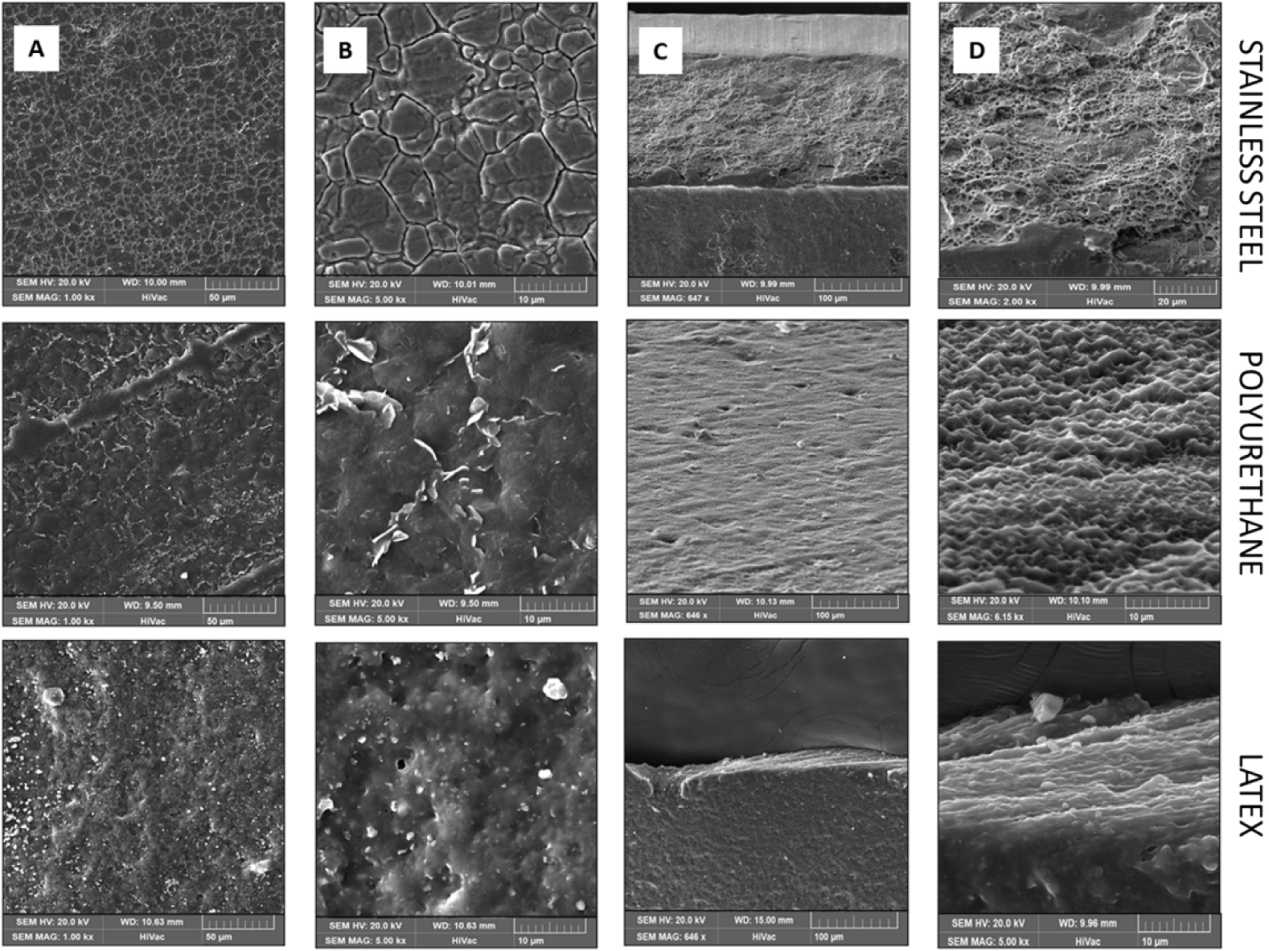
SEM images of stainless steel, polyurethane, and latex surfaces, A and B top view of surface, C and D side view of the coupons.

### Surface Morphology of Food-Contact Materials

Scanning electron microscopy was used to examine the surface morphology of stainless steel, polyurethane, and latex coupons. Representative SEM images are shown in Figure 4A–D.

Stainless steel surfaces exhibited pronounced grooves, crevices, and irregular features distributed across the surface (Figure 4A). These surface characteristics are typical of mechanically processed stainless steel and may provide protected microenvironments that facilitate bacterial retention.^44^ Polyurethane surfaces appeared smoother and more uniform, with fewer pronounced surface defects and a more continuous surface structure (Figure 4B). The reduced number of crevices on polyurethane surfaces suggests fewer protected attachment sites compared to stainless steel surfaces.

Latex surfaces exhibited the smoothest morphology among the evaluated materials, with a largely homogeneous surface and minimal visible surface disruptions (Figure 4C). Despite this smooth appearance, latex surfaces still supported substantial bacterial attachment in subsequent experiments, indicating that surface morphology alone does not dictate bacterial adhesion behavior.

Side-view SEM images of the coupon edges revealed additional material-dependent differences (Figure 4D). Stainless steel edges exhibited rough and irregular fracture features, whereas polyurethane and latex coupons maintained smoother and more uniform edges following cutting. These irregular edge features may provide localized retention sites for bacterial cells, particularly under conditions involving mechanical stress or agitation.

Surface roughness and topographical complexity have been reported to influence bacterial adhesion by increasing the available attachment area and shielding cells from detachment forces.^44^ However, the observations in Figure 4 indicate that surface morphology alone does not fully explain the differences in bacterial attachment observed among the tested materials. Consequently, surface morphology must be considered together with wettability (Table 1) and surface free energy (Table 2) when interpreting material-dependent biofilm behavior.

### Release Behavior of *trans*-Cinnamaldehyde from TC@ZIF-8 Nanoparticles

The release behavior of *trans*-cinnamaldehyde (TC) from 0.5TC@ZIF-8 nanoparticles was evaluated to characterize antimicrobial availability under conditions relevant to surface-based applications. Release experiments were conducted in phosphate-buffered saline (PBS) containing 5% methanol at 19 and 37 °C, representing room temperature conditions and the optimal growth temperature of *Escherichia coli*, respectively. The cumulative percentage of TC released over time is presented in Figure 5.

**Figure 5:**
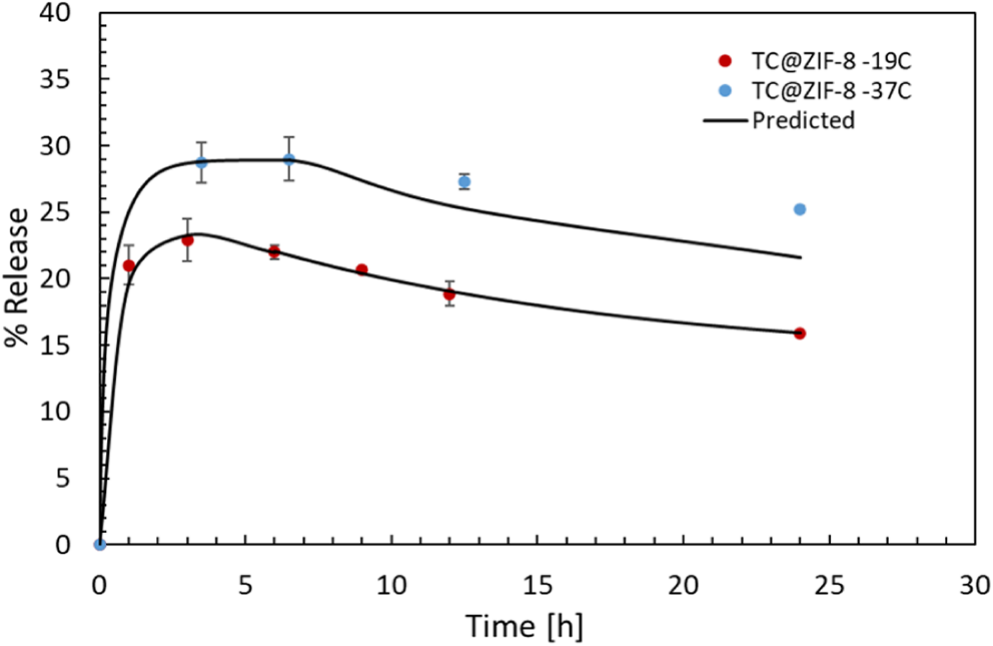
TC % release from 0.5TC@ZIF-8 nanoparticles in PBS: MeOH (5%) medium at different temperatures.

TC release at both temperatures followed a biphasic profile consisting of an initial rapid increase in detectable TC followed by a gradual decline over the remainder of the incubation period. The extent of TC release was greater at 37 °C than at 19 °C, indicating that temperature influenced both framework degradation and compound diffusion. The enhanced release observed at elevated temperature is consistent with increased molecular mobility and accelerated breakdown of the ZIF-8 structure in aqueous environments.

The initial increase in detectable TC is attributed to partial degradation of the ZIF-8 framework in phosphate-containing media, which facilitates the liberation of TC located near the particle surface or weakly retained within the framework pores. The subsequent decrease in detectable TC concentration over time is likely associated with interactions between released Zn^2+^ ions and phosphate species in PBS, resulting in the formation of insoluble zinc phosphate phases.^45^

Degradation of ZIF-8 materials in phosphate-rich environments and the formation of zinc phosphate species have been reported previously and support this interpretation.^45^

To quantitatively describe the observed release behavior, the TC release profiles were fitted using a biexponential kinetic model consisting of a burst release phase followed by an exponential decay phase. The burst release phase and subsequent decay phase were described using eqs 1 and 2:

1 $$\mathrm{Burst}\mathrm{release}\mathrm{phase}:R_{1}\left(t\right)=R_{0}\times \left(1-\exp\left(-k\times t\right)\right)$$

2 $$\mathrm{exponential}\mathrm{decay}\mathrm{phase}:R_{2}\left(t\right)=R_{1}\times \exp\left(-k_{2}\times \left(t-t_{c}\right)\right)$$

where *R*_1_ *(t)* is the percentage of TC released at time *t* during the burst phase, *R*_0_ is the maximum fraction released during this phase, *k* is the burst-release rate constant, *R*_2_ *(t)* is the percentage released at time *t* during the exponential decrease phase, *R*_1_ is the percentage release remaining after the burst phase, *k*_2_ is the rate constant for the exponential decrease, and *t*_c_ is the transition time from the burst phase to the exponential decrease phase. The complete release behavior was described by the combined biexponential model shown in eq 3:

3 $$\mathrm{Combined}\mathrm{model}:R\left(t\right)\{R_{0}\times \left(1-\exp\left(-k\times t\right)\right)R_{1}\left(t_{c}\right)+R_{1}\times \exp\left(-k_{2}\times \left(t-t_{c}\right)\right)$$

Model fitting parameters derived from this analysis are summarized in Table 3. High coefficients of determination (*R* ^2^ > 0.98) were obtained for both temperatures, indicating excellent agreement between the experimental data and the proposed kinetic model. The biexponential release behavior demonstrates that ZIF-8 functions not only as a stabilizing carrier for TC but also as an active participant in the release process through controlled framework degradation.

**Table 3:** TC Release Equations Obtained from Biexponential Model

T^a^[^o^C]	Equation		*R* ^2^
19	BRP^b^	23.4051×(1−exp(−1.8229×t)	0.98
	EDP^c^	$13.7155+8.3683\times (\exp\left(-0.0748\times \left(t-t_{c}\right)\right)$	0.99
37	BRP	13.1635×(1−exp(−9.4342×t)+15.7661×(1−exp(−1.3921×t))	0.99
	EDP	$29.9791\times (\exp\left(-0.0136\times \left(t-t_{c}\right)\right)$	0.98

a *T* = temperature.

b BRP = burst release phase.

c EDP = exponential decrease phase.

From an application perspective, the biphasic release behavior observed for 0.5TC@ZIF-8 nanoparticles is advantageous for surface-based antimicrobial use. The initial release phase provides rapid antimicrobial availability, while the sustained phase supports prolonged exposure to TC.

### Antibacterial Activity of 0.5TC@ZIF-8 Nanoparticles

The antibacterial activity of 0.5TC@ZIF-8 nanoparticles against *Escherichia coli* MG1655 was evaluated by determining the minimum inhibitory concentration (MIC) and minimum bactericidal concentration (MBC). Bacterial growth curves generated in the presence of increasing nanoparticle concentrations are shown in Figure 6.

**Figure 6:**
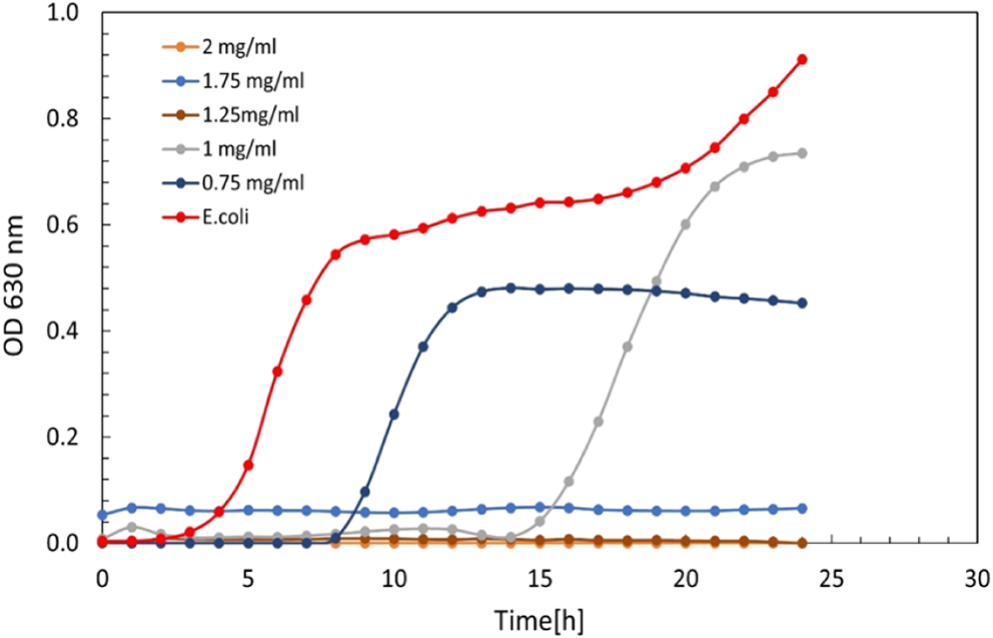
Growth curves of *E. coli* MG1655 in the presence of 0.5TC@ZIF-8 nanoparticles.

Untreated control cultures exhibited typical growth behavior characterized by a short lag phase followed by rapid exponential growth. Exposure to TC@ZIF-8 nanoparticles resulted in concentration-dependent alterations in bacterial growth. At sub-MIC levels, treated cultures exhibited prolonged lag phases prior to entering exponential growth, indicating interference with early adaptation processes. At concentrations corresponding to the MIC, bacterial growth was completely suppressed over the 24-h incubation period (Figure 6). Plate enumeration confirmed bactericidal activity at concentrations equal to or greater than the MBC, consistent with established definitions of antimicrobial efficacy.^32,33^ These findings demonstrate that encapsulation of *trans*-cinnamaldehyde within the ZIF-8 framework preserved antimicrobial activity while enabling sustained inhibitory effects under aqueous conditions. The sustained growth inhibition observed suggests that controlled release from the nanoparticle carrier contributes to prolonged antimicrobial activity.

### Effect of 0.5TC@ZIF-8 Nanoparticles on Bacterial Attachment and Growth of *E. coli* MG1655 on the Test Food-Contact Surfaces

In this part of the study, the attachment and growth of *E. coli* MG1655 on stainless steel, polyurethane, and latex coupons were evaluated in the presence of various concentrations of 0.5TC@ZIF-8 nanoparticles. Additionally, the impact of 0.5TC@ZIF-8 nanoparticles on preformed *E. coli* biofilms on these surfaces was investigated (Figure 7 and Table 4).

**Figure 7:**
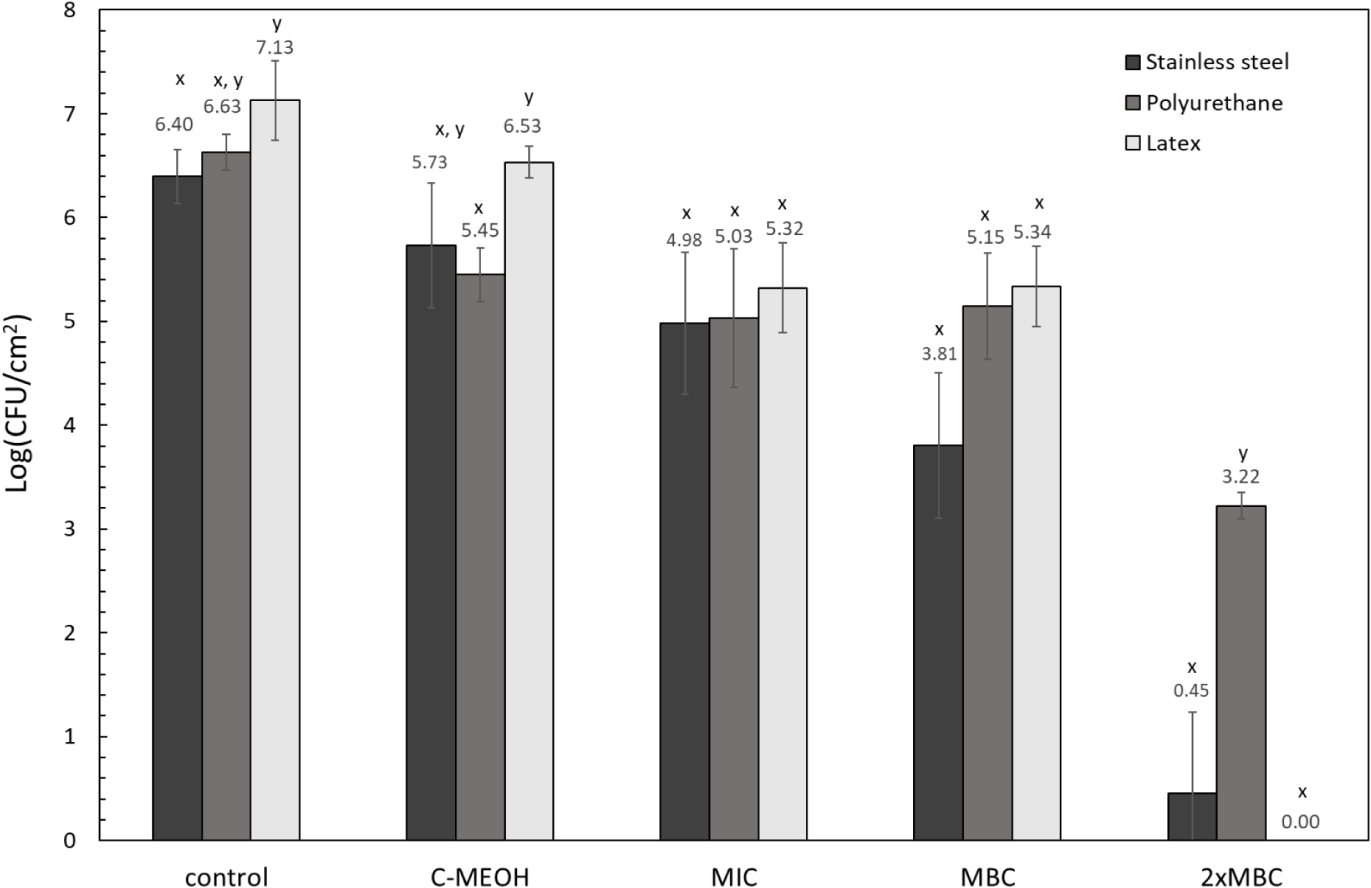
Bacterial attachment and growth inhibition of *E. coli* MG1655 on stainless steel, polyurethane, and latex surfaces in the presence of 0.5TC@ZIF-8 nanoparticles.

**Table 4:** Bacterial Attachment and Growth of *E. coli* MG 1655 on Stainless Steel, Polyurethane, and Latex Surfaces in the Presence of 0.5TC@ZIF-8 Nanoparticles, Log CFU/cm.^2^

	Stainless steel^a^	Polyurethane^b^	Latex^c^
Control	_*x*_ 6.396 ± 0.258^c^	_x,y_ 6.633 ± 0.173^c^	_*y*_ 7.130 ± 0.383^c^
C-MeOH	_x,y_ 5.731 ± 0.603^c^	_*x*_ 5.450 ± 0.259^b^	_*y*_ 6.532 ± 0.151^c^
MIC	_*x*_ 4.981 ± 0.683^c^ ^b^	_*x*_ 5.032 ± 0.663^a^ ^b^ ^c^	_*x*_ 5.322 ± 0.431^b^
MBC	_*x*_ 3.807 ± 0.698^b^	_*x*_ 5.149 ± 0.509^a^ ^b^ ^c^	_*x*_ 5.338 ± 0.385^b^
2xMBC	_*x*_ 0.452 ± 0.783^a^	_*y*_ 3.224 ± 0.128^a^	_*x*_ 0.000 ± 0.000^a^

a Values given are averages of three replicate samples ± standard deviations.

b Means within a column, which are not followed by a common superscript letter, are significantly different (*p* < 0.05). ^x,y^ Means within a row, which are not followed by a common superscript letter, are significantly different (*p* < 0.05).

In control treatments lacking nanoparticles, latex surfaces exhibited the highest levels of bacterial attachment, followed by polyurethane and stainless steel surfaces (Figure 7 and Table 4).

This trend is consistent with differences in surface wettability and surface free energy among the tested materials (Tables 1 and 2).

When TC@ZIF-8 nanoparticles were present during incubation, bacterial attachment decreased in a concentration-dependent manner across all surfaces. At concentrations corresponding to the MIC, bacterial attachment was reduced compared to the control, although measurable bacterial populations remained on all three surfaces (Figure 7).

At 2× MBC, bacterial counts on stainless steel and latex surfaces were reduced to near or below the detection limit, while polyurethane surfaces retained detectable bacterial populations.

These material-dependent differences in attachment inhibition are consistent with variations in surface morphology (Figure 4), wettability (Table 1), and surface free energy (Table 2).

Overall, the results suggest that surface roughness negatively affected bacterial attachment,^44–49^ while high surface free energy and wettability promoted bacterial attachment.^44^

### Removal of Established *Escherichia coli* Biofilms by 0.5TC@ZIF-8 Nanoparticles

The effectiveness of 0.5TC@ZIF-8 nanoparticles for removing established *Escherichia coli* MG1655 biofilms from stainless steel, polyurethane, and latex surfaces was evaluated using 24-h-old biofilms (Figure 8).

**Figure 8:**
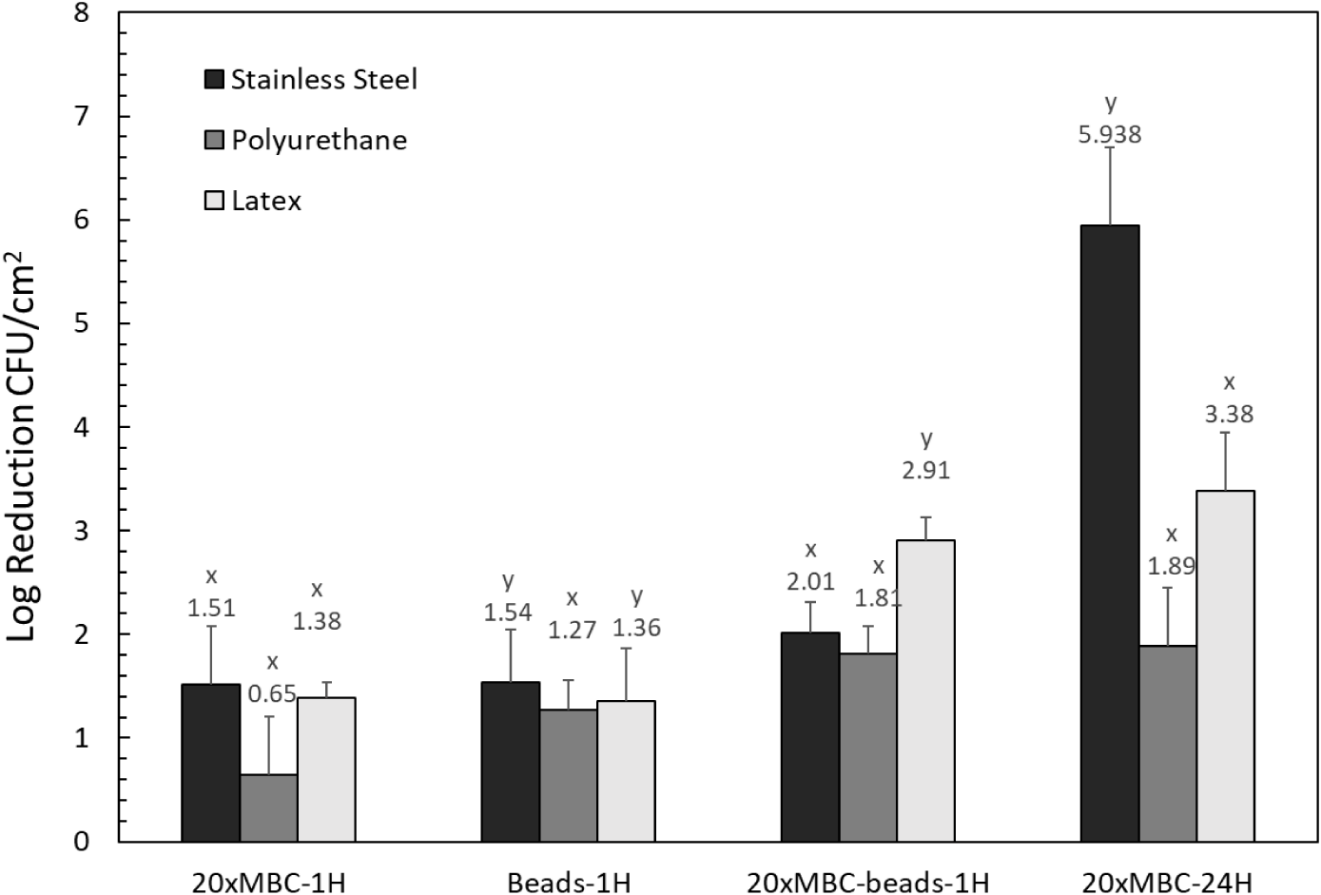
Log reduction on *E. coli* MG1655 population on stainless steel, polyurethane, and latex surfaces after 0.5TC@ZIF-8 treatment.

Short-term exposure to 0.5TC@ZIF-8 nanoparticles resulted in limited biofilm removal across all tested surfaces. In contrast, extended exposure times and higher nanoparticle concentrations produced substantial reductions in surface-associated bacterial populations. Stainless steel surfaces exhibited the greatest biofilm reduction under extended treatment conditions (Figure 8). This enhanced removal may be influenced by the greater surface roughness and edge irregularities observed for stainless steel (Figure 4A,D), which could facilitate nanoparticle penetration into biofilm structures.

The inclusion of mechanical agitation significantly enhanced biofilm removal on all tested materials (Figure 8). Mature biofilms are known to exhibit increased resistance to antimicrobial agents due to the presence of extracellular polymeric substances, often requiring combined chemical and mechanical approaches for effective disruption.^34,35^

Comparison of bacterial attachment inhibition and biofilm removal results demonstrates that preventing initial bacterial attachment requires substantially lower nanoparticle concentrations than those required to remove established biofilms (Figure 7, Table 4, and Figure 8).

## Conclusions

While the synthesis and physicochemical properties of TC@ZIF-8 were previously reported, the present study provides the first systematic evaluation of its effectiveness against surface-adhered biofilms on industrially relevant food-contact materials with distinct surface energies and roughness profiles.

This study demonstrates that *trans*-cinnamaldehyde encapsulated within ZIF-8 nanoparticles is an effective antimicrobial system for controlling *Escherichia coli* MG1655 attachment and biofilm formation on food-contact materials with differing surface properties. Surface wettability, surface free energy, and surface morphology collectively influenced bacterial behavior and the efficacy of nanoparticle-based interventions.

Latex surfaces, characterized by high wettability and surface free energy, supported the greatest bacterial attachment but also exhibited strong responsiveness to nanoparticle-mediated inhibition during early attachment stages. Stainless steel surfaces benefited from both moderate surface energy and greater surface roughness, which enhanced biofilm removal under prolonged treatment conditions. In contrast, polyurethane surfaces showed reduced bacterial attachment but also lower susceptibility to antimicrobial inhibition, underscoring the importance of surface-specific considerations.

The biphasic release behavior of *trans*-cinnamaldehyde from 0.5TC@ZIF-8 nanoparticles enabled rapid initial antimicrobial availability followed by sustained exposure, supporting both attachment inhibition and extended antimicrobial action. While lower nanoparticle concentrations were sufficient to prevent early bacterial attachment, removal of established biofilms required higher concentrations, longer exposure times, and mechanical action, highlighting the resilience of mature biofilm structures.

Collectively, these findings emphasize the value of nanoparticle-based delivery systems combined with surface property analysis for developing effective biofilm control strategies. The results provide insight into the design of targeted, surface-aware sanitation approaches and support the potential application of TC@ZIF-8 nanoparticles as natural antimicrobial agents for food safety and surface hygiene applications.
